# Unique aspects of transcriptional regulation in neurons – nuances in NFκB and Sp1-related factors

**DOI:** 10.1186/1742-2094-6-16

**Published:** 2009-05-18

**Authors:** Xianrong R Mao, Andréa M Moerman-Herzog, Yuzhi Chen, Steven W Barger

**Affiliations:** 1 Department of Anesthesiology, Washington University School of Medicine, St Louis MO 63110, USA; 2 Department of Geriatrics, University of Arkansas for Medical Sciences, Little Rock AR 72205, USA; 3 Department of Neurobiology and Developmental Sciences, University of Arkansas for Medical Sciences, Little Rock AR 72205, USA; 4 Geriatric Research Education and Clinical Center, Central Arkansas Veterans Healthcare System, Little Rock AR 72205, USA

## Abstract

The unique physiology and function of neurons create differences in their cellular physiology, including their regulation of gene expression. We began several years ago exploring the relationships between the NFκB transcription factor, neuronal survival, and glutamate receptor activation in telencephalic neurons. These studies led us to conclude that this population of cells is nearly incapable of activating the NFκB that is nonetheless expressed at reasonable levels. A subset of the κB cis elements are instead bound by members of the Sp1 family in neurons. Also surprising was our discovery that Sp1 itself, typically described as ubiquitous, is severely restricted in expression within forebrain neurons; Sp4 seems to be substituted during neuronal differentiation. These findings and their implications for neuronal differentiation – as well as potential dedifferentiation during degenerative processes – are discussed here.

## NFκB induction pathways

NFκB consists of two subunits of Rel-family proteins, which include RelA (p65), RelB, cRel, p50, and p52 in mammalian cells [1]. RelA, RelB, and cRel subunits contain a transactivation domain; p50 and p52 do not. These subunits can form about a dozen different homo- and heterodimers. The best-characterized dimer is RelA/p50, also known as the canonical NFκB. Rel family proteins have a nuclear localization sequence (NLS) that permits their translocation to the nucleus upon activation, where they bind specific DNA sequences. The consensus bound by canonical NFκB is typically represented by GGGRNNYYCC; other dimers have slightly different preferences [2]. NFκB activation is transient in most scenarios and terminated through the interaction between NFκB and the inhibitory κB proteins (IκBs) and also through degradation by proteasomal activity in the nucleus [3]. IκBs can mask the NLS of Rel proteins, and one of them (IκBα) has a nuclear export sequence (NES) through which the NFκB/IκBα complex is efficiently shuttled back to cytosol, restoring the inactive state. NFκB activation typically results from the breakdown of IκB proteins, an event requiring multiple steps. Once a stimulus activates the pathway, IκB kinases IKK1, IKK2, and IKK3 (the last is a scaffolding protein) are activated, phosphorylating IκBs [4]. Phosphorylated IκBs are degraded through the ubiquitin/proteasome pathway. The degradation of IκBs liberates NFκB and completes the activation cycle.

Despite the similarities between Rel family members, specific components of the NFκB system play unique and nonredundant roles [5,6]. One aspect of this specificity is the preference of various dimers for different DNA sequences [2]. There is also specificity in the stimuli and mechanisms of activation. Tumor necrosis factor-α (TNFα) and other activators of canonical NFκB signal through IKK2. Lymphotoxin-α (a.k.a., "TNFβ") and CD40 ligand, on the other hand, induce the noncanonical pathway via IKK1. IKK2 and canonical NFκB orchestrate inflammation and a few other immune responses; they also promote viability through the induction of several anti-apoptosis genes [7-9]. IKK1 appears to be involved in feedback inhibition of inflammation and contributes critically to development, with particular importance for limb formation [10,11]. The noncanonical pathway utilizes p100 (the precursor of p52) to mask the NLS of RelB, excluding RelB/p100 from the nucleus without involvement of a canonical IκB. Inactive RelB/p100 is converted to active RelB/p52 by IKK1 and NFκB-inducing kinase (NIK). Activated NIK preferentially phosphorylates IKK1 rather than IKK2, and activated IKK1 subsequently phosphorylates p100, which is then partially degraded to p52 through a ubiquitin-dependent mechanism. The resulting RelB/p52 heterodimer constitutes active, noncanonical NFκB.

## NFκB activation in neurons? The case of glutamate

Glutamate is the major excitatory neurotransmitter in the central nervous system (CNS), but it also exhibits toxicity to most types of neurons. This excitotoxicity has been implicated in many disease states [12], and NFκB has complex roles in cellular viability and death [2]. Therefore, elucidation of the potential interactions between glutamate and NFκB is extremely important for understanding both normal and pathological brain functions.

Although NFκB activation is transient in all other systems, it was initially reported to be constitutively active in cortical neurons [13]. Shortly thereafter, that laboratory and others reported that NFκB activity was activated after glutamate treatment in cerebellar granule cell cultures [14,15]. But the neuronal population in cerebellar granule cell cultures cannot be enriched with mitotic inhibitors [16,17]; so these results, obtained from cultures maintained in the presence of serum, raise the question of whether the detected NFκB activity was truly from neurons or from contaminating glia.

Because of our interest in both glutamatergic stimuli and CNS transcriptional regulation, we made extensive attempts to reproduce the activation of NFκB by glutamate. These efforts have routinely utilized a rat cerebral culture system [see Additional file 1] that is almost completely devoid of nonneuronal cell types. Using cell type-specific immunocytochemistry, we determined that mitotic inhibitors were not sufficient for producing glia-free cultures of neurons; nor could a serum-free medium alone achieve this goal. By using a mitotic inhibitor *together with *serum-free medium we found we could restrict glial contamination to less than one percent of cells. Mitotic inhibitors are rather stressful to the neurons themselves; and the most commonly used, cytosine arabinoside (AraC), has been reported to either stimulate [18] or inhibit [19] NFκB itself. Therefore, we employed a transient AraC treatment, including it only in the first four days in culture.

**Figure 1:**
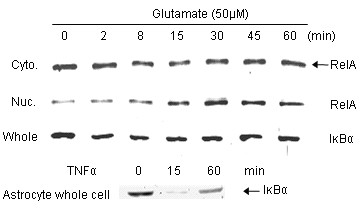
**RelA nuclear translocation in neurons after glutamate treatment**. Nearly pure neocortical neurons were treated with glutamate (50 μM) for the indicated times and cells were harvested for fractionation (**Cyto**.: cytosolic fraction; **Nuc**.: nuclear fraction); these were probed by western blot for RelA. Whole-cell lysates were also prepared from either astrocyte or neuron cultures; these were probed by western blot for IκBα (**Whole**: neuron whole cell lysate). TNFα was applied to the astrocytes at 100 ng/ml for the indicated times (min).

Many of the original reports of NFκB activation in neurons were based on nuclear translocation of the transcription factor as an endpoint. We tested this parameter in our highly enriched cortical neuron cultures. Cells treated with glutamate and then lysed and separated into a cytosolic fraction (supernatant) and a nuclear fraction (whole nuclear pellet sonicated) for analysis by western blotting (note: this is different from the nuclear extract used in EMSA, which only includes the salt-extractable components). Glutamate was indeed found to evoke nuclear translocation of RelA under these conditions (Figure 1). This is consistent with previous findings we obtained with immunocytochemistry [20]. Translocation of RelA-containing NFκB complexes is typically dependent upon degradation of an IκB, so we assessed the levels of IκBα in whole-cell lysates obtained after an identical glutamate treatment. No significant levels of other IκB proteins can be detected in cultured cortical neurons, although multiple antibodies have been tested for each. Interestingly, only a small reduction of IκBα levels could be observed in neurons (Figure 1). This is particularly apparent when compared to the nearly complete loss of IκBα we observed in astrocytes treated with TNFα (Figure 1).

Nuclear translocation is necessary but not sufficient for NFκB activation of transcription. Numerous circumstances have been documented in which NFκB moves to the nucleus without effecting transactivation [21-29]. We began to assay pure neuronal cultures by EMSA and found that NFκB activity was undetectable by this measure after glutamate treatment [30]. Many attempts have been made subsequently, and we have broadened our tests of NFκB activity to include transcriptional activation, as well. No NFκB activity has been detected in glutamate-treated neurons examined by EMSA, reporter-gene transfection, or a stably integrated reporter transgene [2,20,30-32].

One possible explanation for discrepancies between our findings and those of others is the composition of our cell cultures. We tested the potential contributions of glia by comparing our essentially glia-free neuronal cultures to neuron-glia cocultures generated by the inclusion of serum. These experiments consistently showed NFκB activation the cocultures [2,20,30,31]. To determine the cell type contributing this NFκB activity, we made use of a transwell coculture model that permitted us to physically separate neurons from glia after glutamate treatment [31]. In this format, we found that NFκB activity could not be induced by glutamate in pure cultures of *either *neurons or glia alone. However, transwell cultures in which glia and neurons were together for two days showed NFκB induction in the glia but not in the neurons. One hypothesis to explain these results is that glutamate-stimulated neurons release a soluble factor that induces NFκB in glia. Alternatively, neurons may chronically release a factor that alters the acute responses of glial NFκB to glutamate. This latter idea is consistent with our data: glutamate was incapable of inducing NFκB in glia if they were placed with neurons only immediately before glutamate stimulation. This result also demonstrates that glia can be the source of NFκB in cocultures even though pure astrocyte cultures do not show a response to glutamate themselves; e.g., Guerrini *et al*. (1995) [15].

The above studies with glutamate and other stimuli were largely conducted in cell culture. While this approach has limitations, its contributions to our understanding of neuronal transcription are important because it is technically impossible to precisely determine both the location and activity level of a transcription factor *in vivo*. The best strategy for doing so involves making an animal transgenic for a β-galactosidase reporter gene driven by a promoter responsive to the transcription factor of interest. Although β-gal histochemistry is not exquisitely quantitative, it nonetheless reports whether there has been some level of transcription. Two such transgenic models suggest that NFκB is constitutively active in neurons [33,34]. Bhakar *et al*. [33] reported that constitutive NFκB activity could be detected by the κB/β-gal reporter transgene, and this activity was required for survival of neurons. However, electrophoretic mobility shift assays (EMSA) conducted by the authors failed to demonstrate DNA-binding activity for NFκB (personal communication), which raises the possibility that the basal activity of this κB/β-gal reporter is driven by other κB-binding factor(s), such as Sp-factors (below). In addition, the transgene integration site in the genome might be critical for this reporter activity, and those circumstances remain undetermined. Nevertheless, no NFκB activity could be evoked by glutamate in these transgenic neurons [2]. In the transgenic system developed by Fridmacher *et al*. [34], the reporter gene was driven by the p105 (p50 precursor) promoter. This promoter was selected for its NFκB-responsiveness, but Sp1-related factors are also involved in regulation of this promoter via κB and non-κB elements [35]. Furthermore, in the initial report creating this p105-promoter/β-gal line, a complex distinct from NFκB was shown to prominently bind this promoter in EMSA [36]. It is now clear that this constitutive κB-binding activity results from Sp1-related factors [32]. Induction of an IκBα super-repressor inhibited expression of the p105-promoter/β-gal reporter, but IκBα also inhibits activity of Sp1-related factors [37], a phenomenon that will become further relevant in discussions below. Fridmacher *et al*. found that induction of the IκBα super-repressor had no effect on neuronal survival under basal conditions, contradicting the results from the transgenic model reported by Bhakar *et al*. Fridmacher *et al*. did not identify NFκB in neurons by EMSA or other immunological methods.

**Figure 2:**
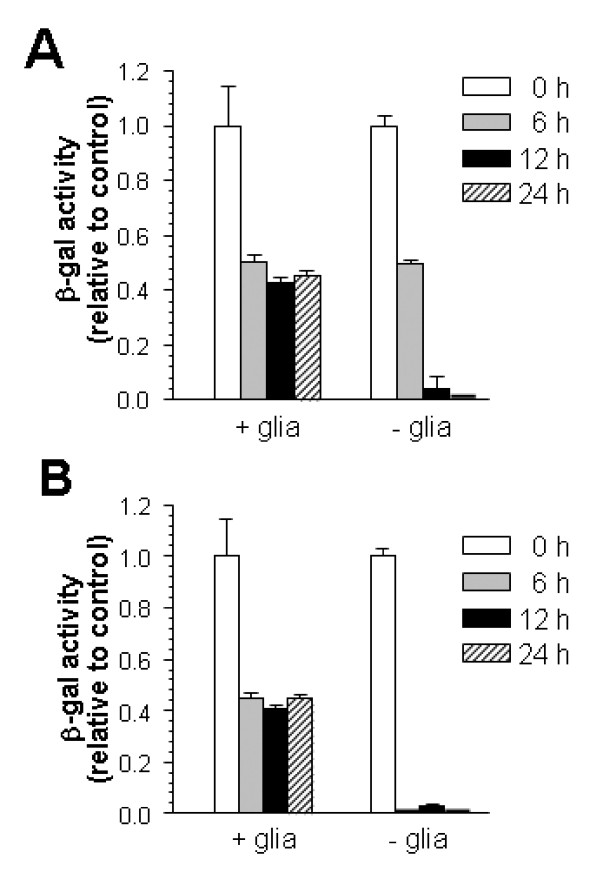
**Glutamate is unable to activate neuronal NFκB in mixed cell cultures**. Neocortical neurons from κB/β-gal transgenic mice were either plated on the top of wild-type astrocytes ("+glia") or retained as nearly pure neuronal cultures ("-glia"). **A**. Glutamate was applied at 50 μM for 10 min, then chased for the times indicated (h). **B**. Glutamate was applied at 20 μM continuously for the times indicated (h). β-gal activity was determined by a luminescent assay. Values represent activity relative to untreated cultures ± SEM in quadruplicate cultures. ANOVA followed by Scheffe post-hoc test showed significance between control and each treatment, and between the "+glia" and "-glia" in all comparisons except the 6 h timepoint in A. (*p *< 0.02).

We have further analyzed *in vitro *neurons from the transgenic line developed by Bhakar *et al*. [33]. The main objective was to explore the possibility that pure neuronal cultures are altered in their handling of NFκB due to the absence of a physical interaction with glia. To test this idea, we cultured neurons from the κB/β-gal reporter line with wild-type glia so that typical neuron-glia interactions could take place, while the reporting of NFκB activity would be specific to the neurons in the coculture. Wild-type astrocytes were first plated and grown to confluency, and then E17 transgenic (κB/β-gal) cortical cells were added one week prior to treatment. The pre-plated wild-type astrocytes suppressed growth of transgenic glia but promoted survival of the κB/β-gal neurons, as histochemistry showed no astrocytes positive for β-gal. These cocultures were compared to cultures of κB/β-gal neurons alone. Two treatment paradigms were tested: 1) a transient exposure to glutamate (10 min) followed by a 6- to 24-hour chase period, or 2) continuous exposure to glutamate for 6 to 24 hours (Figure 2). Once again, neuronally localized NFκB activity could not be induced by glutamate. Indeed, basal β-gal levels were suppressed by glutamate exposure, an effect that was partially suppressed in the presence of glia, presumably due to the ability of astrocytes to metabolize glutamate. This negative effect of glutamate on κB-dependent transcription is consistent with copious EMSA data showing that glutamate reduces the levels of κB-binding Sp1-related factors in neurons (below).

Others have confirmed a similar degree of recalcitrance of NFκB in CNS neurons [38,39]. But contradictory findings have been reported, as well. Most notably, Kaltschmidt and Kaltschmidt [40] provided immunocytochemical data indicating that a five-minute exposure to kainate triggered an increase in "active" RelA immunogenicity (some of which was nuclear) in neurons an hour later. This apparent activation did not occur if a coverslip plated with astrocytes was placed over the neurons after the stimulus. Astrocytes exhibit high rates of glutamate uptake and amination; thus, they would be expected to attenuate events dependent upon glutamate receptors if the agonist was glutamate itself. Indeed, we found that astrocytes inhibited the glutamate-evoked diminution of transactivation in κB/β-gal neurons (Figure 2). However, kainate is not a substrate for glutamate transporters, and no coverslip-borne astrocytes were present when Kaltschmidt and Kaltschmidt applied kainate to their neurons. Their results thus indicate some influence of astrocytes on neuronal RelA after glutamate receptor activation had ceased. This does not appear to be the result of a soluble factor, as the control neurons received astrocyte-conditioned medium. Nor does it appear to depend on cell-cell contact, as the astrocytes on the coverslip were physically separated from the neurons. Notably, the "neuronal" cultures (lacking the astrocyte coverslip) in these experiments were plated in the presence of 10% serum and thus contained approximately 10% glia [40]. It is somewhat surprising that this more intimate association of glia with the neurons was insignificant compared to that provided by the coverslip's astrocytes. In the end, the results of this study remain unexplained, irrespective of their relationship to our results with cocultures. Moreover, Kaltschmidt and Kaltschmidt failed to demonstrate DNA binding or transcriptional activation by NFκB in their cocultures.

Although our original focus was to test the reported induction of NFκB by glutamatergic agonism, we have also examined other stimuli. To date, we have failed to detect appreciable NFκB activity in neurons treated with TNFα, nerve growth factor, brain-derived neurotrophic factor, cholinergic or adrenergic agonists, cytoskeletal drugs, or oxidants (e.g., hydrogen peroxide). Many of these stimuli generated detectable NFκB activity when as few as 5% of the cells in the culture were glia.

Together, these data and their caveats provide the conclusion that no convincing evidence supports the full activation of NFκB in neurons, although the initial stages up through nuclear translocation have been detected by ourselves and others (Figure 1; see also Ref. [20]). Most of our work has been conducted with cultured cortical neurons equivalent to post-natal days 2–5, and it is possible that they fundamentally differ from cortical neurons *in situ *in the adult. However, such cultured neurons are post-mitotic by virtue of our transient AraC treatment, and they exhibit mature responses to glutamate [41]. Thus, they can be said to differ more from the nonneuronal cells that do exhibit NFκB activity than from the mature neurons of adult brain. It is also meaningful that the NFκB function does not appear to be required for normal development or function of the CNS. These parameters are normal in the various mouse lines with genetic ablation of key components of the NFκB pathway, most of which appear to have unique, nonredundant roles [42]. These data strongly suggest that either NFκB is passively defective in neurons or there exist special circumstances actively blocking glutamate-triggered NFκB activity. Either case would be consistent with the notion that the CNS, as a partially immunoprivileged site, requires the silencing of NFκB in neurons. In all, these issues stimulate interest in potential mechanisms that might be responsible for unique regulation of NFκB in neurons.

## Dissecting the neuronal NFκB pathway

### Cloning of RelA and IκBs from neurons

To test if the NFκB pathway is intact in neurons, it would be reasonable to start with RelA. Several RelA splicing variants have been reported [43-47]. One of them (p65^Δ^) is particularly interesting because this RelA splicing variant lacks nine residues critical for DNA-binding [44]. This alternative splicing naturally occurs in human and mouse [45]. If this were the sole splice variant expressed in neurons, this could explain the observed deficiency of NFκB activity in neurons. However, the alternative splice-acceptor site present in human and mouse does not exist in the rat genome. Therefore, a p65^Δ ^would be extremely unlikely in rat neurons. Moreover, we cloned RelA cDNA from cortical neurons (accession # AY307375) and found that all the serine residues potentially critical for RelA function are present. Furthermore, when it was transfected in neurons, rat neuronal RelA showed high activity (see below). These data indicate that there is no inherent defect in rat neuronal RelA. These data also indicate that RelA overexpressed from a transgene can behave differently from its endogenous counterpart, presumably because the transfected RelA is expressed at levels high enough to overwhelm some intrinsic inhibitory process. In addition, these data suggest that caution should be used in interpreting data obtained from NFκB transfection studies (e.g., Ref. [48]).

Glutamate treatment triggers a relatively small reduction of IκBα proteins, suggesting that the unresponsiveness of neuronal NFκB might result from a defect in IκB degradation. IκBα degradation is triggered by phosphorylation of two serine residues, an event which signals degradation through a ubiquitin-dependent pathway involving β-TrCP [49]. Either an alteration of those critical serines or a deficiency in β-TrCP-dependent ubiquitin could result in insufficient IκB degradation and low NFκB activity. We cloned IκBα and IκBβ genes from rat neurons, and their sequences showed no mutations compared to those published (data not shown). In addition, expression of a β-TrCP transgene did not permit induction of a κB-driven luciferase reporter-gene construct (below). Therefore, both RelA and major IκB genes appear normal in neurons. We extended our scope to other players in the pathway in a quest for potential differences between CNS neurons and other cell types.

### Profiling components of the NFκB pathway in neurons

**Figure 3:**
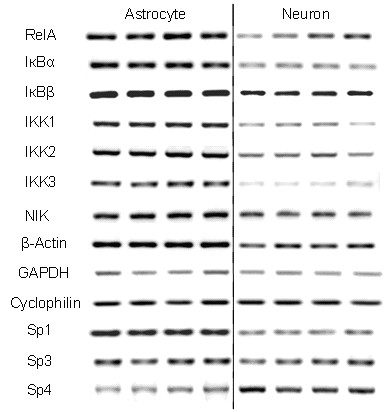
**Differential levels of mRNA for key components of the NFκB pathway in neurons versus glia**. RNA was harvested from rat primary neocortical neurons and astrocytes. Semiquantitive RT-PCR was performed on equal amounts of total mRNA to survey expression of genes in the NFκB pathway, housekeeping genes, and Sp-family factors [see Additional file 1]. Each lane represents one of four individual cultures.

RT-PCR was used to probe the mRNA expression levels of the major components of NFκB pathway. After optimizing PCR conditions, unequivocal products for each of these genes were obtained from glia. Then, identical conditions were used to compare glia and neuron samples. In each case, more than one pair of primers was used, and consistent results were observed for the same gene [see Additional file 2]. The results from one set of primers for each gene are shown (Figure 3). The genes tested included RelA, IκBα, IκBβ, IKK1, IKK2, IKK3/NEMO, and NIK. Controls included three house-keeping genes: β-actin, glyceraldehyde-3-phosphate dehydrogenase (GAPDH), cyclophilin. For reasons explained below, we also examined the Sp-factors Sp1, Sp3, and Sp4. Generally, the mRNA level of every component in the NFκB pathway was expressed significantly lower in neurons than in astrocytes (*p *< 0.05 for each gene, astrocytes vs. neurons). β-actin was also expressed at a lower level in neurons (*p *< 0.05). However, GAPDH, cyclophilin, and Sp3 mRNA levels were almost equivalent in neurons and astrocytes; Sp4 mRNA levels were significantly higher in neurons than in astrocytes (*p *< 0.05) (see also Ref. [50] for Sp-factor expression). Therefore, these PCR results appear to reflect a significantly lower expression in neurons than in glia for every component of the NFκB pathway.

### Reporter assays for complementing the NFκB pathway in neurons

**Figure 4:**
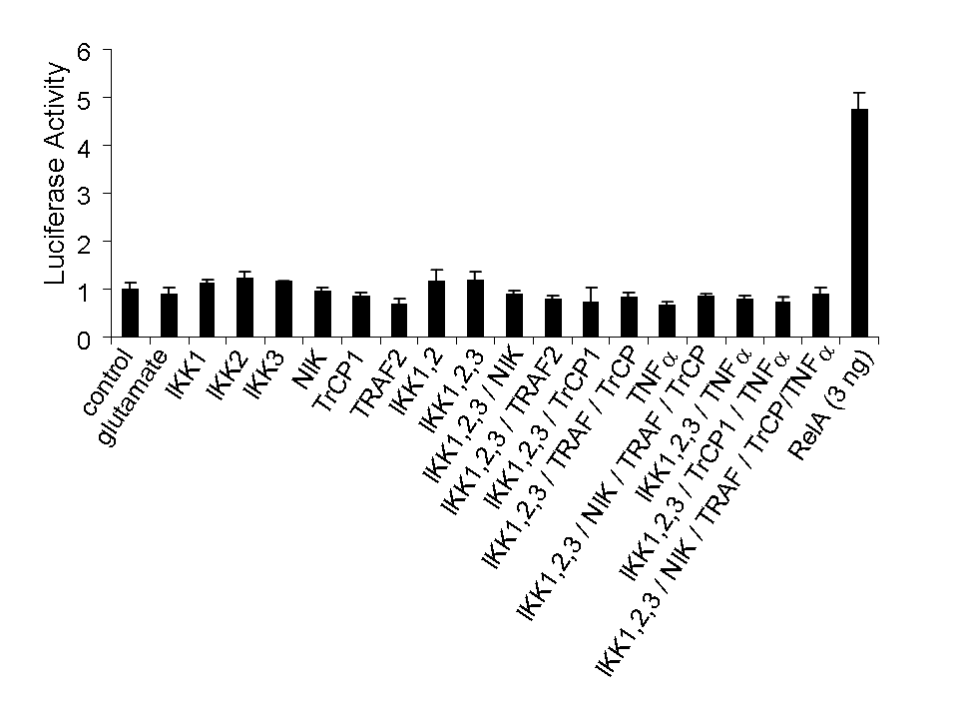
**NFκB activity in neurons cannot be evoked by supplementing key components of its pathway**. IKK1, IKK2, IKK3, NIK, β-TrCP1, and TRAF2 were transfected either alone or in combination as indicated to test NFκB activity in neocortical neurons by reporter assays with a κB-driven firefly luciferase construct. Some transfection conditions were also further treated with rat TNFα (1–100 ng/ml). Rat RelA transfection served as a positive control. After 24 h, firefly luciferase activities were determined relative to *Renilla *luciferase reference. Values reflect the mean ± SEM of triplicate cultures. Except RelA transfection, no condition created a significant change compared to control.

**Figure 5:**
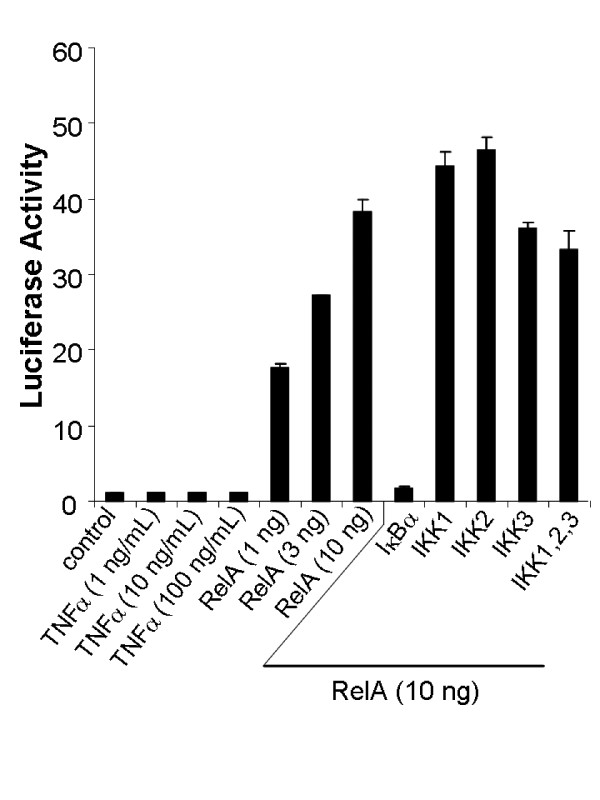
**Upstream components of NFκB pathway cannot boost exogenous RelA activity in neurons**. Primary neocortical neurons were transfected with a κB-driven firefly luciferase reporter, along with RelA and other key factors in the NFκB pathway. (The dose of RelA plasmid was selected to be intermediate with respect to the maximal level of the assay's dynamic range.) In some conditions, cells were treated with rat TNFα (10 ng/ml) after transfection. Relative luciferase activity from RelA (100 ng) transfection was 111.05 ± 8.9 (mean ± SEM) (not shown). All plasmids were 100 ng/well, except those labeled otherwise. After 24 h, firefly luciferase activities were determined relative to *Renilla *luciferase reference. Values reflect the mean ± SEM of triplicate cultures. *p < 0.05 (t-test; other significant differences are not labeled).

Another intriguing fact is that Sp1 protein is nearly absent in neurons, although Sp1 mRNA can be detected [51]. Similarly, the deficiency of NFκB mRNAs in neurons may actually under-represent a more profound deficiency in protein levels. This issue was addressed through a reconstitution approach using expression of a κB-driven luciferase reporter-gene as the endpoint. It should be noted that the κB element in our luciferase reporter excludes nucleotides that allow binding by Sp-factors and RBP-Jκ (below), thus rendering it specific for NFκB. Several components of the NFκB pathway, including β-TrCP, were overexpressed by transfection [see Additional file 1] to test whether their expression would be permissive for the induction of endogenous NFκB activity in neurons. Overexpression of a TNF receptor association factor (TRAF) is sufficient to activate NFκB in many cell types [52]. However, neither TRAF2 (Figure 4) nor TRAF6 (not shown) could activate NFκB in neurons, as measured by a cotransfected κB-driven luciferase reporter. Indeed, none of the constituents of the NFκB pathway was effective. Several components were cotransfected in combination with one another (Figure 4). Even so, NFκB activation was never detected in any of these conditions except for one: the overexpression of RelA. A low dose of RelA expression plasmid was used to replace TRAF2 to repeat the experiment. This modest level of exogenously supplied activity could not be boosted further by cotransfection of any of combination of IKKs (Figure 5). These findings suggest that there are no passive functional deficiencies of major components of the NFκB pathway in neurons.

## Searching for mechanisms of NFκB repression

All of the requisite components of the NFκB system appear to be expressed in forebrain neurons (Figure 3 and data not shown). In addition, providing additional components by transfection did not create a responsive system in neurons. These findings are consistent with there being an active repression of NFκB in forebrain neurons. To test candidate mechanisms for this repression, we used the following approaches:

1) Transcription coordinator p300 is important for NFκB transactivity, and p300 might be competed out by some other factors such as estrogen receptor [53] in neurons. However, overexpression of p300 in neurons with or without glutamate treatment did not increase κB-driven reporter activity.

2) Acetylation has been reported to be important for RelA function [54]. A triple mutation of RelA (K_218/221/310_→R) completely incapable of being acetylated was transfected into neurons. This RelA mutant possessed activity as high as the wild-type RelA transgene, suggesting that acetylation is not essential for RelA activity in neurons.

3) Phosphorylation has been reported to be crucial for RelA function in some scenarios. Many serine residues have been identified as important: Ser_529 _[25], Ser_536 _[55], Ser_337 _[56], Ser_205_/Ser_278_/Ser_281 _[57], and Ser_311 _[58]. RelA cloned from rat cortical neurons did not show mutations at any of the relevant serines (see below). Moreover, these phosphorylation events affect RelA's transactivation activity rather than its DNA-binding activity, and neuronal NFκB seems to be deficient in DNA-binding activity. Finally, we tested a S_281_→G RelA mutant in reporter assays, and this mutant functioned as well as the wild-type RelA transgene. Therefore, it seems highly unlikely that phosphorylation of RelA is a critical determinant of NFκB activity in neurons.

4) Transcription factor p53 has been reported to suppress NFκB activity [59]. However, we found that p53 inhibitor pifithrin-α had no effect on NFκB reporter-gene activity in neurons.

5) Another Rel-factor RelB has been shown to inhibit RelA activity by disrupting its DNA binding [60]. However, when RelB was transfected into neurons, it did not alter the activity of co-transfected RelA. Moreover, RelB expression levels are very low in neurons as determined by several antibodies in western blot analyses.

6) The progesterone receptor can be induced to bind directly to NFκB and inhibit its activity, and progesterone is a component of the medium we routinely use for neuronal cultures: Neurobasal with B27 supplement. To control for progesterone and any unknown components of Neurobasal/B27, we tested for NFκB in neurons cultured in minimal essential medium containing N2 supplements with or without progesterone [20]. Again, reporter-gene assays and EMSAs showed no basal nor induced NFκB activity in neurons.

## Binding of κB enhancers by Sp1-related factors

Specific protein-1 (Sp1) is the prototypical member of a group of highly homologous zinc-finger transcription factors that includes Sp1 through -9 and the Krüppel-like factors (of which there are as many as 17 in humans) [61,62]. Despite similarities in the zinc-finger domains among the family, only Sp3 and Sp4 share the DNA binding sequence first identified with Sp1. Like most NFκB target sequences, the sites bound by these three Sp-factors are GC-rich. We and others have reported specific, robust, and functional binding of Sp1, -3, and -4 to a subset of NFκB target sequences [32,63]. Indeed, binding by the latter two accounts for more than 95% of the binding to certain κB elements in neurons.

In our initial examination of Sp-factors in neurons, we assumed that reports of Sp1 ubiquity in the CNS were accurate. This prejudice, combined with antibodies that were at least partially cross-reactive with multiple Sp-family members, led us to conclude that it was indeed Sp1 that was responsible for κB binding in EMSA with neuronal extracts. Subsequently, we found that the presence of Sp4 in mature neurons corresponds to a near absence of Sp1 [51]. Immunofluorescence data obtained in our cultures, as well as in the cortex of adult rats, reveals negligible colocalization of Sp1 with neuronal markers and clear colocalization of Sp1 with the astrocyte marker glial fibrillary acidic protein (GFAP); the converse is true for Sp4. We also assessed the expression of these proteins in cell cultures highly enriched for neurons or glia, as well as in glial and neuronal cell lines. Both western blot analysis and RT-PCR analysis of mRNA indicate that Sp1 expression is much higher in glia than in neurons, and Sp4 shows the converse. Finally, we performed supershift analyses in EMSA; Sp1 antibodies altered the mobility of the Sp-factors only in glial cultures and Sp4 antibodies did so only in neuronal cultures. By all these methods Sp3 was found to be expressed at similar levels in both neuronal and nonneuronal cells.

The replacement of Sp1 by Sp4 appears to have important ramifications for gene expression. Sp1 is primarily a transcriptional inducer, but Sp4 has often been connected to transcriptional repression, particularly when expressed in the presence of Sp3 [64,65]. Although others have shown that Sp1 overexpression in neurons can have powerful biological effects, our data would argue that the natural situation is replacement of this transcriptional inducer by the repressor-competent Sp4. While Sp4 can act as a transcriptional activator [66], it appears incapable of doing so in the presence of Sp3 [32,64], which is abundant in cortical neurons [51]. Alternatively, Sp4 may be primarily a stimulatory factor at "Sp1" binding sites and inhibitory at κB elements. Further studies are required to explore these interesting possibilities.

**Figure 6:**
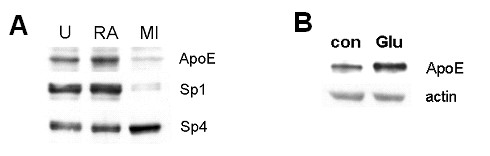
**ApoE declines with Sp1 during neuronal differentiation but is induced by glutamate**. **A**. NTera2 cells were harvested undifferentiated (U) or after incubation as neurospheres in retinoic acid for 14 days (RA). Subsequent to RA treatment, additional cultures underwent selection by incubation with a cocktail of mitotic inhibitors (MI) for 7 days. Equal amounts of protein were resolved by SDS-PAGE, and the levels of ApoE, Sp1, and Sp4 were assessed by western blot analysis. **B**. Differentiated NTera2 cells were untreated or treated with 20 μM glutamate for 20 h, then levels of ApoE and actin were assessed by western blot.

Sp1 and Sp4 dynamics may have important effects on the terminal differentiation of post-mitotic neurons. Indeed, Sp1 plays key roles in mitosis [67]. It is responsible for inducing expression of key cell-cycle regulatory genes such as cdc25C [68], cyclin B1 [69], cyclin D1 [70], cyclin E1 [71], and thymidine kinase [72]; these genes may be much less responsive to Sp4, a trait already demonstrated for thymidine kinase [72]. Sp1 is phosphorylated by cyclin-dependent kinase 2 (Cdk2) at Ser_59 _during the S-phase of the cell cycle, and this results in transactivation of dihydrofolate reductase (DHFR) [73], an essential event in the production of thymidine for DNA synthesis. Sp1 is also associated physically with cyclin A and cyclin E1 during S-phase [74]. Reductions in the level or activity of Sp1 can block mitosis [75,76]. We have noted a dramatic suppression of Sp1 expression after differentiation of NTera2 cells into neurons (Figure 6A).

Sp4 expression, already apparent in undifferentiated NTera2 cells [51], appears to increase slightly during the differentiation process (Figure 6A). It is possible that induction of Sp4 is an important event in readying neural stem cells for differentiation down the neuronal path. The functional implications of the Sp1:Sp4 ratio may not be limited to cell cycle regulation. Apolipoprotein E (ApoE) expression is normally limited to astrocytes and not neurons [76], and the ApoE promoter is responsive to Sp1 [77]. We found that during differentiation of NTera2 cells [see Additional file 1], ApoE levels decreased proportionally with Sp1 while Sp4 remained abundant (Figure 6A). This is consistent with a prior demonstration that ApoE is squelched during differentiation of NTera2 cells [78] and may indicate that Sp4 exerts a transcriptional repression at the Sp1 element in the ApoE promoter.

Neuronal expression of ApoE can be induced by excitotoxic stress [79,80], and we found that glutamate-mediated excitotoxic stress results in an increase in ApoE levels in differentiated NTera2 cells (Figure 6B; see also Ref. [81]). The induction of neuronal ApoE expression by glutamate and other excitotoxic agents may involve release from the repressive influence of Sp4. The DNA-binding activity attributable to Sp3 and Sp4 is diminished by glutamate treatment [30-32,51]. This phenomenon occurs rapidly – within 30 min of treatment of CNS neurons destined to die 20–24 hours later [30,51]. We determined that this depletion is dependent on calcium influx through NMDA receptors and correlates with the appearance of smaller DNA-binding proteins. The levels of intact Sp3 and Sp4 also decrease with similar kinetics and pharmacology in western blot analysis [51]. It was eventually determined that activation of the calcium-dependent protease calpain is responsible for the loss of these proteins (and their conversion to smaller intermediates that retain DNA-binding activity). Rapid loss of Sp-factors is also associated with delayed toxicity caused by oxidative stress [30], though this apparently occurs through a calpain-independent process because it does not generate the same proteolytic products as does glutamate (data not shown). It is not known whether the early loss of Sp3 and Sp4 contributes to the cell death occurring later; it is possible that there are compensatory benefits resulting from attenuation of the levels and activities of these transcription factors. The latter idea is given some support by the finding that neuronal Sp-factors have an inhibitory influence on transcription via a regulatory element in the *Sod2 *gene (below). Thus, their degradation under conditions of stress could be permissive for the elevation of a protective antioxidant enzyme.

## Cell-type specificity of a κB element: regulation of the *Sod2 *gene

To test the effects of Sp-factors on κB-driven genes in neurons, we analyzed several κB sequences predicted to bind Sp-factors [50]. One of these lies in the gene for superoxide dismutase-2 (Mn-SOD), which has an intronic enhancer element previously shown to be regulated by NFκB in nonneuronal cells lying within rat *Sod2 *intron 2 (RSI) [82,83]. We confirmed that the RSI κB element was bound by NFκB using EMSA. In nuclear extracts from TNFα-treated astrocytes, three major complexes were detected [50]. Through supershift assays with an array of antibodies against Rel-factors and Sp-family proteins, the three complexes were identified as p50/p50, p65/p50, and Sp1 comigrating with Sp3. On the other hand, there was only one complex from neuronal nuclear extracts, and it was identified as Sp3 comigrating with Sp4 [50]. These data indicated that the proteins capable of binding the *Sod2*-κB site differed dramatically between astrocytes and neurons; notably, binding by NFκB and Sp1 was absent in neurons.

To test the functional consequences of distinct *Sod2*-κB-binding proteins, a luciferase reporter plasmid was constructed containing 500 bp of the RSI. Again, no activation could be detected in neurons after glutamate treatment [50]. Furthermore, Sp3 and Sp4 suppressed the promoter activity through the κB site in neurons, because expression was elevated by either point mutation of the κB site or depletion of available Sp-family proteins with decoy oligonucleotides. As predicted by the EMSA data, IκBα cotransfection had no effect in neurons. RelA cotransfection significantly enhanced the reporter activity, which could be completely blocked by IκBα coexpression. The activity of the intronic promoter was high in astrocytes (at least partially due to the fact that glia are activated by transfection itself) and was drastically inhibited by Sp1-decoy oligonucleotides or IκBα; activity was completely abolished by combination of the two [50]. Together, these data showed that Sp-factors (Sp3 and Sp4) are the major neuronal κB-binding factors and that they suppress transcriptional activity by interacting with the intronic κB site in neurons. On the contrary, glial Sp-factors (Sp1 and Sp3) result in activation that can synergize with that produced by NFκB through the same κB site. A tandem κB-element has been identified in the promoter for βAPP [84]. Mutation of these two κB sites in the βAPP promoter (3.8 kb) did not change its activity in neurons, further demonstrating that NFκB is not involved in regulating κB elements in neurons under basal conditions.

## Significance for neuroinflammatory disease

Because of its importance for expression of cytokines and other factors related to inflammation, NFκB has been targeted for inhibition by some therapeutic strategies. The actions of certain pharmacotherapeutic agents such as aspirin are thought to include inhibition of NFκB, and this may be an effect of more recently developed drugs such as agonists of peroxisome proliferator-activated receptors (PPAR) [85,86]. Therapeutic objectives also inspired the development of specific IKK inhibitors, which has been a boon to research, as well. With the discovery of anti-apoptotic actions of NFκB, some have supposed that its inhibition might be dangerous to CNS neurons [87]. While our findings do not rule out a contribution of NFκB to neuronal survival under every condition, they do suggest that control of inflammation by inhibition of NFκB will not cause significant levels of neuronal cell death. Recently, it has been proposed that homo- or heterodimers of c-Rel are neuroprotective whereas p50/RelA is neurotoxic [88]. However, c-Rel-containing species are activated via the canonical IKK2-dependent pathway and are thus just as sensitive to salicylates [89] (and other IKK inhibitors). Salicylates and IKK inhibitors are associated with positive outcomes in a variety of neurotoxicological models [90-94], indicating that neurons probably do not depend upon tonic NFκB activity.

**Figure 7:**
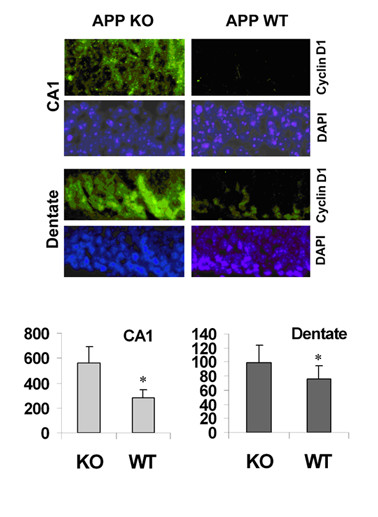
**Induction of neuronal expression of cyclin D1 in βAPP-deficient mice**. Brain sections from wild-type ("WT") and βAPP-knockout ("APP-KO") mice were subjected to immunofluorescence detection of cyclin D1 (green). The sections were counterstained with DAPI to visualize nuclei (blue). Representative images are shown. The pixel intensity of specific staining was randomly sampled in the CA1 and dentate cell body layer. The mean values and standard errors are shown in graphically. Statistical comparison between the two genotypes by t-test indicated *p *< 0.05 (n = 4) in each hippocampal region.

As a specific neuroinflammatory condition, Alzheimer's disease might hold relevance for the unique expression and regulation of transcription factors we have found in neurons. We recently conducted an analysis of βAPP expression in normal aging and Alzheimer's disease [81]. These studies documented a glutamate-triggered induction of βAPP expression that is dependent upon the product of the ε3 allele of apolipoprotein E (ApoE3). (Possession of the major alternative allele in humans, ApoE4, is associated with a dramatically increased risk for Alzheimer's disease.) Moreover, our findings indicated that this glutamate→ApoE3→βAPP axis becomes uncoupled in the early stages of Alzheimer pathogenesis. ApoE levels continue to rise with advancing Alzheimer pathology, but *de novo *expression of βAPP in neuronal somata is dramatically depleted. To test the consequences of this loss of βAPP, we examined βAPP-knockout mice [95]. Neurons in the brains of these mice showed elevated expression of cyclins D1 (Figure 7) and E1 (not shown), which have promoters that are responsive to Sp1. Such ectopic expression of cell cycle markers in post-mitotic neurons is a curious component of pathology found in Alzheimer's and other neurodegenerative conditions and one that appears to trigger apoptosis [96]. It is possible that this phenomenon involves derangement of the Sp4:Sp1 ratio in stressed neurons.

**Figure 8:**
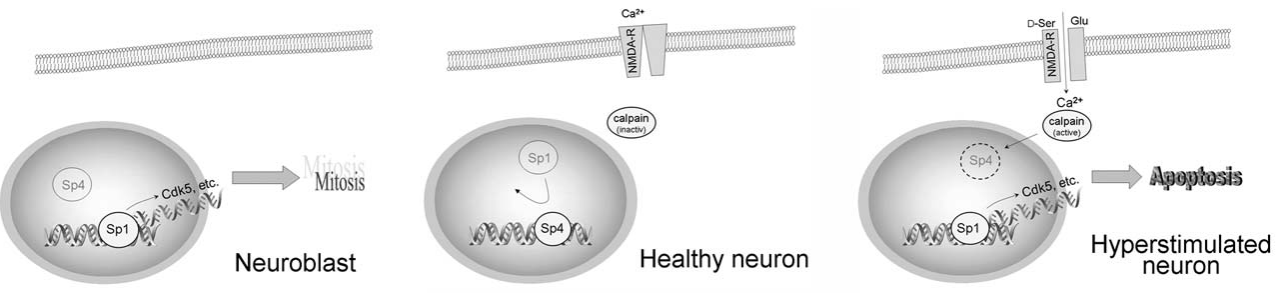
**Schematic for the roles of Sp1 and Sp4 in neuronal differentiation and degeneration**. Immature neuroblasts appear to similar levels of Sp1 and Sp4, perhaps competing for regulatory control of genes not strongly related to differentiated neuronal function. After differentiation, mature neurons have much lower levels of Sp1; Sp4 competitively inhibits DNA binding and exerts transcriptional repression on genes related to mitosis and other genes inappropriate for neurons, such as ApoE. Upon excessive stimulation of glutamate receptors, a large calcium influx activates calpain, which digests Sp4. This may relieve the transcriptional repression of mitotic genes, permitting their inappropriate expression in terminally differentiated neurons; the latter appears sufficient to trigger apoptosis.

In light of the information produced by our analysis of Sp1-related factors, we propose a model with implications for neurodegeneration such as that occurring in Alzheimer's (Figure 8). The ApoE gene has an Sp-factor binding site in its promoter that permits induction by Sp1 in nonneuronal cells [77], but ApoE is suppressed in neurons [97]. We propose that this is a result of the Sp1→Sp4 switch during neuronal differentiation. Moreover, we propose that the induction of ApoE by glutamate we have observed [81] results from a calpain-mediated degradation of Sp4, thus liberating ApoE from an Sp4-mediated suppression. Finally, we propose that dysregulation of normal aspects of neuronal differentiation in Alzheimer's, perhaps brought about by loss of Sp4, leads to ectopic expression of cell cycle proteins and neuronal cell death.

## Competing interests

The authors declare that they have no competing interests.

## Authors' contributions

XM generated most of the data and wrote the first draft of the text. AMM-H generated data on expression of Sp1, Sp4, and ApoE during differentiation of NTera2 cells and contributed substantially to the text. YC generated data on the expression of cyclin D1 in APP-knockout mice. SWB conceived of most of the studies and contributed substantially to the text.

## Supplementary Material

Additional file 1 **Supplemental methods**. The file contains detailed methodology for the experiments presented in this review. Click here for file

Additional file 2 **Table S1**. This table provides details about PCR primers and conditions and is cited in Additional File 1. Click here for file
