# Supplementary material for: Unique aspects of transcriptional regulation in neurons – nuances in NFκB and Sp1-related factors
Source: J Neuroinflammation. 2009 May 18;6:16. doi: 10.1186/1742-2094-6-16 (PMC2693111; doi:10.1186/1742-2094-6-16)
Supplement: Additional file 1 — Supplemental methods. The file contains detailed methodology for the experiments presented in this review. [file 1742-2094-6-16-S1.doc]

**Supplemental Methods**

**Cell cultures.** Primary neuronal cultures were established from the neocortices of 18-day Sprague-Dawley rat fetuses or 17-day mouse fetuses. Pregnant females were euthanized by an overdose of isoflurane followed by decapitation. Fetuses were removed sterilely and decapitated. Brains were removed in a HEPES-buffered Hank’s balanced salt solution lacking calcium and magnesium and supplemented with 10 μg/ml gentamycin (HBH). The cerebral hemispheres were removed and stripped of meningeal tissue. The hippocampus was removed, and the remainder of the cerebrum was utilized for neocortical cultures. Tissue was incubated at room temperature for 7 min with 1 mg/ml trypsin in HBH, washed with HBH, and incubated another 12 min with 1.5 mg/ml soybean trypsin inhibitor in HBH. The tissue was again washed with HBH, resuspended at 333 μl per brain, and then dissociated through successive trituration with a 10-ml pipette followed by a Pasteur pipetted fire-polished to reduce its bore size to approximately half the original. The cell suspension was plated at ~1x10^5^/cm^2^ in precoated tissue culture plates. Plates were prepared by coating with 100 μg/cm^2^ polyethyleneimine (PEI) in borate buffer, washing with phosphate-buffered saline (PBS), and then incubating overnight in minimal essential medium with Earle's salts (MEM, Life Technologies; Carlsbad CA) supplemented with 10% heat-inactivated fetal bovine serum (FBS) and 14.75 mM additional KCl. After 2 h to allow adherence, the cells were washed to Neurobasal medium containing 2% B27 supplement (both from Life Technologies), 0.5 mM L-glutamine, and 10 μg/ml gentamycin. AraC (3-10 µM, optimized by batch) was added for the first five days, then removed by replacement of the medium with fresh Neurobasal/B27/L-glutamine/gentamycin. Neurons were utilized after 8-10 days in culture.

Astrocyte cultures were established from the cerebrums of Sprague-Dawley rats or C57BL/6 mice on postnatal day 0-1. Neonates were anesthetized by hypothermia and decapitated. The brains were removed in HBH, and the cerebral cortices were separated, stripped of meningeal tissue, and minced to ~3.3 mm^3^ with a scalpel. Tissue was incubated at room temperature for 7 min with 1 mg/ml trypsin in HBH, washed with HBH, and incubated another 12 min with 1.5 mg/ml soybean trypsin inhibitor in HBH. The tissue was again washed with HBH, resuspended at 700 μl per brain, dissociated by trituration with a 10-ml pipette, and plated at a rate of one brain per T75 tissue culture flask. The culture medium was MEM supplemented with 10% FBS and 10 μg/ml gentamycin. After 10-12 days, the majority of microglia were removed by vigorous agitation and lavage with complete culture medium; the remaining cells had the appearance of type-I astrocytes.

The NTera2 human neural-lineage teratocarcinoma cell line was obtained from Stratagene (La Jolla CA) and routinely maintained in MEM supplemented with 10% FBS. For differentiation, trypsinized cell suspensions were incubated at least 14 days in 100-mm bacteriological plates at 1 x 10^6^ cells/plate in MEM/FBS containing 10 μg/ml retinoic acid; fresh medium was exchange every 2-3 days. The nonadherent aggregates (“neurospheres”) formed under these conditions were dissociated by trypsinization and plated into PEI-coated dishes as for primary neurons (above) in MEM/FBS. For further selection (“RA” in Fig. 6), cultures were incubated an additional 10 days in MEM/FBS supplemented with 40 μM AraC to and 4 μM; fresh medium containing these mitotic inhibitors was exchanged every 2 days.

**Transient transfection and luciferase activity assay*.*** Neurons plated in 24-well plates were transfected with Lipofectamine 2000 according to the manufacturer’s (Life Technologies) instructions. Two reporter constructs were used: 100 ng/well κB-reporter (pNFκB-Luc; Panomics, Fremont CA) and 50 ng/well of pCMV-RL (Promega, Madison WS), the latter expressing the *Renilla* luciferase used to standardize transfection efficiency. The κB-reporter contains six tandem repeats of a κB site that is not bound by Sp1-related factors (GGGAATTTCC). In some cases, plasmids expressing other proteins—i.e., RelA—were added at 100 ng (unless otherwise indicated). The DNA in a given reaction was raised to a final of mass of 1 μg via addition of pBluescript (Statagene, San Diego CA) and then combined with 2 μl Lipofectamine 2000 in 50 μl MEM. After a 15-min incubation, this mixture was added into the existing culture medium. After 3 h, the cultures were washed thrice with MEM and returned to Neurobasal/B27/glutamine. The day after transfection, some cultures were treated with glutamate or TNFα (Peprotech Inc., Rocky Hill NJ). Cells were harvested one day after treatment, and luciferase activities were determined by a dual-luciferase assay kit (Promega) using a Veritas microplate luminometer (Turner BioSystems, Sunnyvale CA). The ratios between firefly luciferase reading (from the κB-reporter) and *Renilla* luciferase (from pCMV-RL) were taken to represent normalized NFκB activities.

**Immunofluorescence.** Mice of the APP-knockout line [95] were backcrossed for 10 generations to C57BL/6. Nullizygous animals and wild-type littermates were anesthetized with isoflurane to effect and decapitated. Brains were removed and immersion-fixed in 4% buffered paraformaldehyde for ~4 h, blocked in coronal planes, and dehydrated through graded alcohols. After paraffin embedding, sections (10 µm) were cut and mounted on subbed slides. Sections were treated with HCl (2N) for 30 min at room temperature, washed five times before blocking in PBS/3% normal goat serum/0.5% Triton X-100 for 10 min, and incubated overnight with mouse anti-cyclin D1 (Neomarker/Thermo Fisher Scientific, Fremont CA) at 1:50. Brain sections without any primary antibody were used as negative controls. Sections were washed twice followed by a 1-h incubation with Alexa Fluor 594 goat anti-mouse secondary antibody (Molecular Probes, Eugene OR) at 1:300. The sections were counterstained with DAPI. Fluorescence images were collected with a CoolSnap camera (Photometrics, Tucson AZ) using a 40x objective on a Nikon Eclipse 600 fluorescence microscope synchronized to MetaView software (version 6.2r2, Universal Imaging Corporation, West Chester PA), which was also used to make measurements of average pixel intensity in randomly selected fields.

**Reverse transcription and polymerase chain reaction (RT-PCR).**  The protocol has been detailed elsewhere [32]. The primers used for PCR are detailed in a supplemental table [see **Additional file 2**]. Annealing temperature for all reactions was 61˚C. The cycle numbers and other conditions were optimized to achieve linear rates of amplification.
