# Supplementary material for: Unique aspects of transcriptional regulation in neurons – nuances in NFκB and Sp1-related factors
Source: J Neuroinflammation. 2009 May 18;6:16. doi: 10.1186/1742-2094-6-16 (PMC2693111; doi:10.1186/1742-2094-6-16)
Supplement: Additional file 2 — Table S1. This table provides details about PCR primers and conditions and is cited in Additional File 1. [file 1742-2094-6-16-S2.pdf]

Table S1. Primers for RT-PCR

Gene	Primers forward / reverse	Cycles	Product (bp)
RelA	CCC CTC CGC CTG ACC C<br>GGC GAT CGT CTG TGT CTG G	29	370
IκBα	CCA CTC CGT CCT GCA GGC C<br>CGT AGG GCA ACT CAT CTT CC	27	391
IκBβ	CTC AAT AAA CCG GAG CCT ACG<br>GCG CTC TGG TTG TCA GGT CTG	30	444
IKK1	GGA ATC CCT GGA ACA GCG TG<br>CGT CTC GCC ATC TTG AGG	29	423
IKK2	GCT GTG TCC TTC AGG AGC C<br>GTC CTT TGG TCT CTT GGC	33	517
IKK3	GAG GGA AGG ATT CGA GCA<br>CCT CAA TCC TGG CTG ACT	9	541
NIK	GAG CCC ATC CAC CGA GCA<br>GCA CGC CGG AAC TCA GGG T	30	555
β-actin	GTC CTC TCC ATG TGG TTT TC<br>GCT GCG CTC TCG TAA TTG TG	21	439
GAPDH	GAA CGG ATT GGC CGT ATC G<br>TGG GGG TAG GAA CAC GGA A	23	692
Cycloph.	CA AGT CCA TCT ACG GAG AGA A<br>GCA ATC CTG CTA GAC TTA AGG	23	433
Sp1	CCC CAG GTG ATC ATG GAG C<br>CTT CTC TCC TGT ATG TCT ACG	30	383
Sp3	CGG AGG GTA GCT TGC ACC TG<br>GGA TGT CTT GAT TGC TGG TGG C	28	446
Sp4	GGG ATG AGC GAT CAG AAG AAG G<br>CCA CCG TCT GAA GTT GGG GG	32	520
